# The Foot’s Arch and the Energetics of Human Locomotion

**DOI:** 10.1038/srep19403

**Published:** 2016-01-19

**Authors:** Sarah M. Stearne, Kirsty A. McDonald, Jacqueline A. Alderson, Ian North, Charles E. Oxnard, Jonas Rubenson

**Affiliations:** 1 School of Sport Science, Exercise and Health, The University of Western Australia, Perth, WA, 6009, Australia; 2 Willetton Podiatry, Willetton, WA, 6155, Australia; 3 School of Anatomy, Physiology and Human Biology, The University of Western Australia, Perth, WA, 6009, Australia; 4 Biomechanics Laboratory, Department of Kinesiology, The Pennsylvania State University, University Park, PA, 16802, USA

## Abstract

The energy-sparing spring theory of the foot’s arch has become central to interpretations of the foot’s mechanical function and evolution. Using a novel insole technique that restricted compression of the foot’s longitudinal arch, this study provides the first direct evidence that arch compression/recoil during locomotion contributes to lowering energy cost. Restricting arch compression near maximally (~80%) during moderate-speed (2.7 ms^−1^) level running increased metabolic cost by + 6.0% (*p* < 0.001, *d* = 0.67; unaffected by foot strike technique). A simple model shows that the metabolic energy saved by the arch is largely explained by the passive-elastic work it supplies that would otherwise be done by active muscle. Both experimental and model data confirm that it is the end-range of arch compression that dictates the energy-saving role of the arch. Restricting arch compression had no effect on the cost of walking or incline running (3°), commensurate with the smaller role of passive-elastic mechanics in these gaits. These findings substantiate the elastic energy-saving role of the longitudinal arch during running, and suggest that arch supports used in some footwear and orthotics may increase the cost of running.

Running has classically been characterized by the spring-mass paradigm^1^,^2^. During running, gravitational potential and kinetic energy is temporarily stored as elastic strain energy, primarily in tendons, during the first half of stance and subsequently returned in the second half of stance, helping to propel the body forward and upward. This form of elastic energy reduces the metabolic cost of running by sparing mechanical work otherwise required by active muscle tissue^3^,^4^.

Ker and colleagues^5^, identified the longitudinal arch of the foot as an elastic storage-return mechanism. These authors estimated, by simulating the loads experienced during running in cadaver feet, that approximately 17% of the mechanical work of running could be stored and returned by the foot’s arch as it undergoes compression and recoil over the stance phase^5^ and that this contributes to the economy of running. This theory has subsequently been adopted in numerous investigations ranging from analyses of running mechanics^6^, the evolution of human running^7^, and footwear design^8^.

Since the initial study by Ker *et al*.^5^ the hypothesis that the foot is an energy-saving spring has never been tested directly during locomotion and it is unknown to what extent the compression, and subsequent storage and return of elastic energy in the foot’s longitudinal arch affects the metabolic cost of locomotion. Here we propose that the metabolic energy saved by the arch spring is a function of the amount of positive mechanical work it supplies passively (non-metabolically) and the cost of performing that work had it instead been done by active muscle requiring metabolic energy.

We test this hypothesis experimentally using custom-manufactured orthotic insoles designed to restrict arch compression and thus reduce arch elastic work. To test our predictions about the effect of arch strain on locomotor cost, we used orthotic insoles to vary arch strain during walking and running at different inclines and foot strike types (rearfoot strike [RFS] vs forefoot strike [FFS]).

Two separate custom insoles were designed for each participant. The first insole was designed to restrict arch compression near-maximally compared to that during shod running (Full Arch Insole; FAI) and the second was designed to restrict compression by approximately 50% during stance (Half Arch Insole; HAI). Despite the nearly two-fold difference in arch compression between the HAI and FAI, we hypothesized that both would result in a comparable reduction in elastic energy storage/return during running and thus a similar increase in metabolic cost. It is expected that relatively little elastic energy will be stored in the first 50% of arch compression based on the non-linear nature of the arch compression-elastic energy relationship identified by Ker *et al*.^5^ (see Online Supplementary Material Fig. S4). Given the modest arch loads at the prescribed testing speed we expected increases in the energy cost of level running of ~10% based on the arch load vs. energy storage data of Ker *et al*.^5^ and our model for metabolic energy expenditure. In this context, the FAI and HAI were designed to test if the predicted increase in metabolic cost was associated specifically with a reduction in the arch elastic energy as opposed to a more general gait modification linked to the degree of arch restriction.

Compared to level running, we predicted that restricting arch compression during walking and incline running would not have as pronounced of an impact on metabolic cost. Walking involves lower loads^9^, is a pendular as opposed to a spring-mass gait, and relies more on the arch windlass mechanism^10^ as opposed to an arch spring mechanism. We consequently hypothesized the arch spring mechanism has a smaller energy-saving effect in walking compared to running and tested this by restricting arch compression during walking using the FAI. During incline running, the loads experienced are similar to level running and the arch is able to store and return elastic energy^11^. However, the additional positive mechanical work production required to raise the center of mass vertically during incline running cannot be generated from previously stored elastic energy^11^. We therefore predicted that the energetic cost associated with limiting the arch spring relative to the total cost of incline running would be smaller than that of level running. Finally, we hypothesized that restricting arch compression would have a greater effect on the energy cost of level running in habitual FFS runners compared with habitual RFS strike runners, suggesting greater reliance on the arch spring for reducing running costs.

To test these questions we measured metabolic cost (oxygen consumption), arch compression using three-dimensional (3D) motion capture, and ground reaction forces and joint kinetics on an instrumented treadmill. Using this data we estimated the elastic energy stored and returned by the arch, the total mechanical work of locomotion and the metabolic cost of restricting arch elastic energy storage/return.

## Results

### Arch Elastic Energy and Metabolic Cost in Level Running

**Figure 1:**
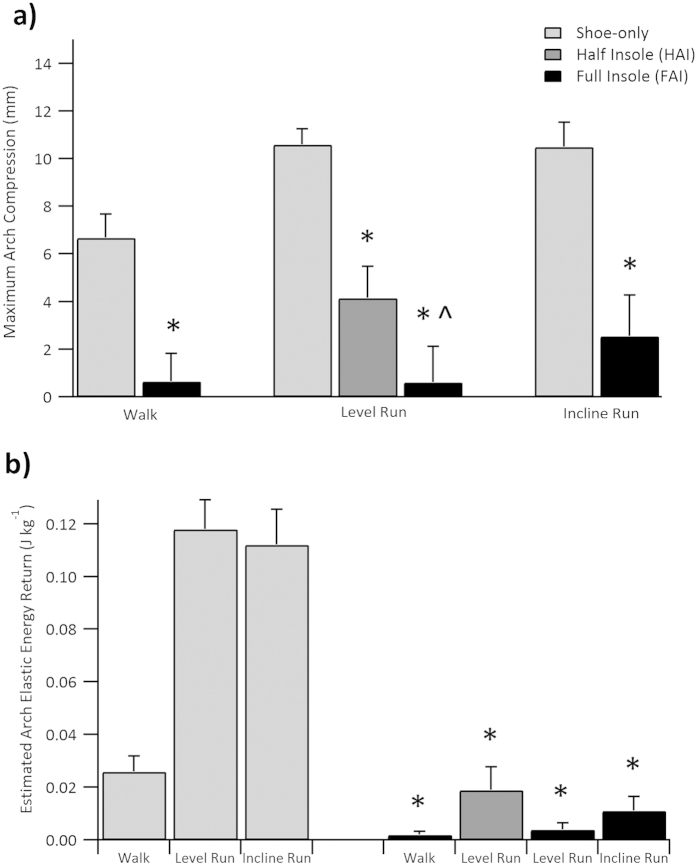
(**a**) Maximum arch compression (mm; mean ± S.E.M.) relative to arch height at minimal shoe-only level running initial foot contact. **(b)** Estimated elastic energy (J kg^−1^, mean ± S.E.M.) returned from the arch of the foot in one step. *indicates significantly different (*p* < 0.05) to the minimal shoe-only trial in the same condition, ^indicates significant difference between the half arch insole (HAI) and full arch insole (FAI) (level running only).

Arch compression (navicular displacement) was significantly reduced in the insole conditions compared with the minimal shoe-only level running. A 61.4 ± 33.4% reduction in arch compression was observed with the HAI (*p* < 0.001, *d* = 1.22) and 78.9 ± 24.7% reduction when the FAI was worn (*p* < 0.001, *d* = 1.46; Fig. 1a and Online Supplementary Fig. S2). Estimated arch elastic energy return per step was also reduced (81.5 ± 26.4% and 97.2 ± 5.4% reductions in the HAI and FAI, respectively; both *p* < 0.001, HAI *d* = 1.53 and FAI *d* = 1.72; Fig. 1b) and per distance traveled (HAI and FAI both *p* < 0.001, HAI *d* = 1.55 and FAI *d* = 1.72; Table 1). FAI arch compression restriction was significantly greater than the HAI condition (*p* = 0.032, *d* = 0.60; Fig. 1a; Table 1) but estimated elastic energy return was not (*p* = 0.121, *d* = 0.58; Table 1).

**Figure 2:**
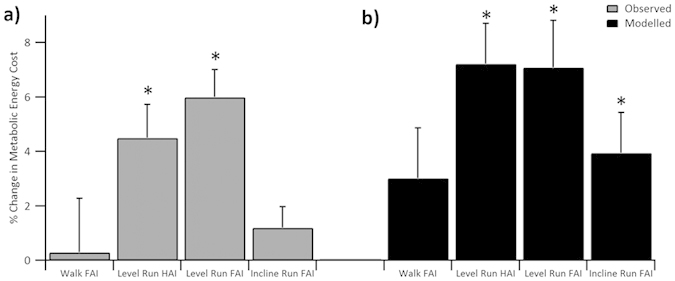
(**a**) Experimentally observed and (**b**) model-predicted percent change in the gross metabolic cost of locomotion (mean ± S.E.M.) from the minimal shoe-only to insole trial across walking, level running and incline running conditions. FAI = full arch insole, HAI = half arch insole. * indicates significant (*p* < 0.05) increase in metabolic energy cost [(**a**) experimental, (**b**) modeled] between the minimal shoe-only and insole trial within the same condition.

The model-predicted increase in metabolic cost of transport (*E*_*arch*_) as a result of reducing arch compression was statistically significant in both the HAI (*p* = 0.001, *d* = 0.77) and FAI (*p* = 0.004, *d* = 0.72) conditions compared with minimal shoe-only level running (Table 1, Fig. 2b). The metabolic cost of HAI and FAI level running resulting from our model were not significantly different from one another (*p* = 0.920, *d* = 0.01; Table 1, Fig. 2b). In line with the modeled metabolic costs, both the HAI and FAI resulted in a statistically significant increase in the experimentally-observed metabolic cost of transport during level running (HAI *p* = 0.012, *d* = 0.53; FAI *p* < 0.001, *d* = 0.67; Table 1, Fig. 2a). As with the modeled costs, there was no statistical difference in the observed experimental metabolic cost between the HAI and FAI conditions (*p* = 0.211, *d* = 0.18). The modeled and experimental metabolic costs were not significantly different from one another for either the HAI or FAI (Table 1). The insoles had a small non-significant effect on total limb mechanical work (ANOVA main effect *p* = 0.121; Table 1).

### Arch Elastic Energy and Metabolic Cost in Walking and Incline Running

Compared to the minimal shoe-only condition, the insole (FAI) reduced arch compression during walking by 82.4 ± 21.1% (*p* < 0.001, *d* = 1.13) and during incline running by 68.5 ± 30.6% (*p* = 0.001, *d* = 1.13; Fig. 1a). The estimated arch elastic energy return per step in both conditions was significantly reduced (96.3 ± 6.9% and 91.6 ± 15.9% reductions per step in walking and incline running, respectively; both *p* < 0.001, walking *d* = 1.12; incline running *d* = 1.53; Fig. 1b). The model-predicted metabolic cost of transport in FAI walking was not statistically greater compared to the minimal shoe-only condition (*p = *0.131, *d* = 0.25; Fig. 2b, Table 1). A statistically significant increase in the modelled metabolic cost of transport was calculated for FAI incline running over the minimal shoe-only condition (*p = *0.025*, d* = 0.59; Fig. 2b, Table 1). The experimentally observed metabolic cost of transport was likewise not affected during walking, nor was it affected in incline running (walking *p* = 0.950, *d* = 0.01; incline running *p* = 0.164, *d* = 0.18; Table 1, Fig. 2a). The insoles had a non-significant effect on the total limb mechanical work of locomotion in walking and incline running (walking *p* = 0.782, *d* = 0.03; incline running *p* = 0.074, *d* = 0.23; Table 1).

### Arch Elastic Energy and Metabolic Cost in Rearfoot vs Forefoot Running

Arch compression in the minimal shoe-only level running condition was significantly greater in FFS compared with RFS runners (FFS 12.0 mm vs RFS 9.2 mm; *p* = 0.022, *d* = 1.10; Table 1). However, the estimated arch elastic energy return (*p* = 0.490, *d* = 0.35), total limb mechanical work (*p* = 0.181, *d* = 0.43) and metabolic cost of transport (*p* = 0.584. *d* = 0.29) did not differ between foot strike groups in minimal shoe-only level running (Table 1). This was also true for FAI conditions between RFS and FFS runners (*p* = 0.483, *p* = 0.092, *p* = 0.568, respectively). Both the modelled and experimental metabolic costs were not statistically different between foot strike groups in any of the HAI and FAI conditions (all conditions *p* > 0.05; Table 1).

## Discussion

By examining the effect of restricting compression of the foot’s longitudinal arch on the metabolic cost of locomotion, the present study provides direct evidence supporting the energy-sparing spring theory of the arch. Our analyses and model generally support the hypothesis that the arch spring saves metabolic energy by reducing the mechanical work that would otherwise need to be generated by active muscle (Fig. 2). Indeed, the elevated metabolic cost of level running after restricting arch compression near-maximally (~80%, FAI) and by ~60% (HAI) was predicted within 1% and 2.5%, respectively, of the measured values based on the cost of replacing lost elastic arch work with muscular work. The agreement between the modelled and experimental effect on the metabolic cost of level running was remarkably robust across a nearly two-fold difference in arch compression (FAI 10 mm reduction in compression vs HAI 6.5 mm reduction; Fig. 1a), strengthening the interpretation that the elevated metabolic cost results specifically from lost arch elastic energy. We propose that energy costs do not show a clearer difference between FAI vs HAI because elastic energy storage increases non-linearly with arch loading^5^, with the majority of the elastic energy stored in the final 25% of arch compression (Online Supplementary Material Fig. S4). Therefore, near equal amounts of arch elastic energy storage/return was removed in both the FAI and HAI conditions (Fig. 1b), resulting in a non-significant difference in the metabolic cost of running (Fig. 2, Table 1).

The estimated reduction in the elastic energy return from the arch in the FAI level running condition equaled 8.8% of the total limb mechanical work of running, similar to the 6.0% increase in the gross locomotor cost (7.4% net 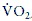). As expected, these values are lower than Ker *et al*.’s^5^ estimate of 17% due to our slower running speed (2.7 m s^−1^ compared to Ker *et al*.’s 4.5 m s^−1^) and therefore lower arch elastic energy storage/release and subsequent smaller arch compressive loads. If the contribution of the arch spring to the total mechanical cost of running increases with speed, as the data from Ker *et al*.^5^ suggest, the arch may have an even more pronounced role in reducing locomotor costs at faster running speeds. What makes the arch-spring such an effective energy-saving mechanism? It is notable that the arch spring, unlike tendon structures (e.g. the Achilles tendon;^12^,^13^), achieves elastic energy recycling largely in absence of muscle activity. The distinction between this passive and other primary active spring mechanisms (e.g. triceps surae/Achilles muscle-tendon-unit) is important since the later requires metabolic energy to maintain tension in the spring (cost of force). In this regard, the arch spring may be the most effective energy saving structure in the lower limb.

Despite confirming that arch compression is greater in FFS compared with RFS runners in the minimal shoe only level run (*p* = 0.022; Table 1), our hypothesis that forefoot runners would be more affected by restricting arch compression was not supported. The lack of difference in the metabolic cost resulting from the FAI between foot strike groups was, however, predicted by our model. The similar model-predicted metabolic costs between FAI running in RFS and FFS arose because of a non-significant difference in the reduction of arch elastic energy storage/return between groups. These findings raise questions regarding differences in arch compliance between RFS and FFS runners, although additional analyses in these groups are required to assess arch material properties in detail.

Our hypotheses that the arch-spring mechanism has a smaller effect on the energy cost of walking and incline running were supported. For walking, the small energetic effect can be explained to a large extent by the smaller role of arch elastic energy storage/return in normal gait (Fig. 1b). It is also possible that the FAI increased midfoot rigidity and thus may have improved the effectiveness of the plantar-flexion torque,^14^,^15^ saving metabolic energy. The percent increase in metabolic cost of FAI incline running computed from our model was smaller than that of FAI level running but was nevertheless significantly greater than the observed experimental effect. We do not have a definite answer to explain the poorer agreement between the increase in metabolic cost and the predicted increase in muscle work during incline FAI running. When running uphill the primary function of muscle is to generate the positive mechanical work of raising the body vertically. Therefore, it remains possible that replacing lost passive-elastic energy recycling may be less important in incline running compared to level running, which follows spring-mass mechanics.

Orthotic insoles and arch-support footwear are occasionally prescribed to runners to alter foot and lower limb biomechanics and tissue loading. The findings of this study suggest that certain arch supports may hinder the arch’s elastic energy storage and subsequently lead to an increase in running energy cost. A number of studies have reported an increase in the energy cost of running when wearing orthotic insoles^16^,^17^,^18^, although this effect may in part be due to added weight^19^. Perl *et al*.^6^ found a statistically significant 3% increase in metabolic cost when participants ran in traditional arch supporting running shoes compared with minimalist shoes, even after controlling for strike technique, shoe mass and stride frequency. The benefits of using corrective orthotics or footwear designed with significant arch support should therefore be weighed against their possible effect on running energetics. In contrast to running, our findings suggest that using rigid supportive shoes or insoles that prevent arch collapse are likely to have little energetic consequence during walking given the smaller reliance on, and reductions in, arch elastic energy storage/return.

Finally, the evolution of the longitudinal foot arch is regarded as a key adaptation for obligate hominin bipedalism^20^,^21^,^22^,^23^,^24^. Although the evolution of the arch is debated, recent fossil evidence suggest that *Australopithecus afarensis* (~3.2 million years) possessed at least a partial longitudinal arch^22^. The functional significance of the longitudinal arch in the evolution of human bipedal gait has often been attributed to the rigid mid-tarsal lever system allowing effective plantar-flexion during toe-off^25^,^26^. A complementary theory surrounding the evolution of the longitudinal arch is that its spring like properties lower the energetic cost of endurance running^5^,^7^. Our study provides support for the arch functioning as both a rigid lever in walking and an energy-sparing spring in running. The insoles had no effect on the metabolic cost of walking despite restricting ~80% of arch compression. The absence of any energetic difference might result, in part, because the insoles enhanced the effect of midfoot rigidity in walking. On the other hand, restricting the arch’s spring function in level running resulted in a clear increase in metabolic cost. Further, that we only observed an energetic consequence of arch restriction during level running and not incline running may offer added insight into the movement behavior and environment of early *Homo*. The landscape inhabited by early *Homo* was invariably not limited to horizontal ground, although given that the arch only provided an energetic advantage during level and not incline running begs questions of how landscape influenced the evolution of the human foot and bipedal gait and how early *Homo* navigated this landscape.

## Methods

### Participants

Eight habitual RFS and nine habitual FFS male runners were included in the study. The participants had not experienced any lower limb injuries in the six months prior to testing nor presented with any pre-existing gait abnormalities. No significant differences in measured physiological variables, as determined by a series of independent t-tests, existed between foot strike groups (age- RFS 25.5 ± 4.4 years, FFS 27.6 ± 3.4 years; height- RFS 185.3 ± 6.9 cm, FFS 181.8 ± 4.8 cm; weight- RFS 79.4 ± 6.8 kg, FFS 75.7 ± 5.9 kg; weekly running distance- RFS 39.4 ± 21.1 km, FFS 42.2 ± 36.0 km; mean ± SD). Participants did not regularly wear prescriptive orthotic insoles. Included participants were deemed to have normal foot structure as determined by the Foot Posture Index^27^ (FPI) (RFS 1.4 ± 1.4, FFS 1.0 ± 2.8). Measured foot variables did not differ between RFS and FFS groups; foot length- RFS 277.0 ± 11.4 mm, FFS 272.4 ± 6.1 mm; resting arch height (from sole to navicular tuberosity)- RFS 50.3 ± 7.3 mm, FFS 49.5 ± 9.5 mm; and Achilles tendon moment arm (perpendicular distance from lateral malleolus to Achilles)- RFS 45.3 ± 4.1 mm, FFS 46.8 ± 5.4 mm. All FPI and foot anthropometric measurements were taken by a single experienced clinician (I.N.). Participants provided written, informed consent prior to inclusion in the study. All procedures were approved by The University of Western Australia Human Research Ethics Committee (Approval ID: RA/4/1/4541) and the study was carried out in accordance with the approved guidelines.

### Custom Arch-restricting Insoles

Two pairs of custom-made foot insoles were manufactured for each participant from 3D scans of the participants’ feet in a non-weight bearing neutral sub-talar joint position (ScanAny, Orthotech laboratories, Blackburn, Melbourne). Both insoles were made with the following specification; four millimeter polypropylene, high density arch fill (shore value ~350–400), four degree intrinsic rear foot grind, a balanced fore foot, maximum arch congruency and the heel ground to less than one millimeter such that heel-toe drop was deemed negligible. One insole was designed to fill the participants arch when the foot was positioned in a neutral non-weight bearing position, theoretically allowing minimal arch compression during locomotion (full arch insole; FAI). The second insole had a peak arch height five millimeters lower than the FAI, with the aim of allowing ~50% arch compression (half arch insole; HAI). The five millimeter reduction was chosen based on pilot work and arch compression data from Perl *et al*.^6^ and Ker *et al*.^5^. Participants were provided the insoles two weeks prior to testing to become familiar with wearing them.

### Testing Conditions

New Balance Minimus road MR00 shoes were provided to all participants to wear for testing (approx. weight 180 grams, zero heel-toe drop, no medial arch support and a uniform EVA midsole). Pockets filled with lead weights were affixed to the laces of both shoes in order to standardize foot weight across all shoe and insole conditions. We chose a minimal shoe as a control condition in order to standardize non-insole effects as much as possible (e.g. effect of shoe sole cushioning^28^). Prior to testing, participants completed a five minute warm-up on a force-plate instrumented split belt treadmill (Bertec Corporation, Columbus OH, USA) at a slow run.

Testing comprised of the following conditions; i) shoe-only walk, ii) FAI walk, iii) minimal shoe-only level run, iv) HAI run, v) FAI run, vi) minimal shoe-only incline run, and vii) FAI incline run. All trials were completed on the force-plate instrumented treadmill and the order of conditions randomized to prevent any fatigue or order effects. To further ensure fatigue and trial order were not influencing results, the first condition was repeated at the end of the testing session. Participants reported minimal discomfort and no conscious change in their running technique whilst wearing the insoles (see Online Supplementary Table S3 for questionnaire results).

All running conditions were performed using the runner’s habitual foot strike technique as confirmed by a sagittal high speed video camera (Casio EXILIM EX-F1, Casio Computer Co. LTD., Shibuya-ku, Tokyo; 300 Hz). In accordance with the literature, a RFS was defined when the heel of the shoe made initial contact with the ground and a FFS defined when the ball of the foot made first contact^29^,^30^. A standardized walking speed of 1.1 ms^−1^ (representing a comfortable speed on the treadmill and within the range of the participants’ pilot tested preferred walking speeds) was selected to minimize arch compression and subsequently elastic energy contribution. To ensure our results were not affected by walking speed, a sub-set of participants (n = 8) also performed the minimal shoe-only and FAI walk at their individually preferred walking speed (average 1.3 ± 0.1 ms^−1^). Similar metabolic cost results were found in the preferred walking speed and the 1.1 ms^−1^ walking trials (13.6 ± 1.3 vs 13.1 ± 1.5 ml kg^−1^ min^−1^, respectively; *p* = 0.190), including a similar minimal effect of the FAI on the metabolic cost of walking. All running trials were performed at 2.7 ms^−1^ (level and incline trials were performed at the same velocity to control for any speed effects). Pilot testing on a sub-set of participants (n = 8) revealed that running at faster speeds (3.5 ms^−1^) caused the insoles to compress and thus limited the effect of the insole on arch compression, likely due to the higher joint loading at this speed. During incline trials the treadmill was set at three degrees (although not specifically instructed to do so, all runners maintained their level habitual foot strike technique). This inclination was selected to increase the mechanical work and metabolic cost but within aerobic levels as faster speed/incline combinations risked reliance on anaerobic metabolic pathways^31^. The chosen running speed thus represents an optimized speed to test the effect of the insoles in both level and incline conditions.

### Metabolic Cost

Participants were asked to abstain from caffeine on the day of the testing and to not eat in the two hours prior to arriving at the laboratory. Expired gasses were collected during rest (standing) and walking/running trials. Participants were required to breathe into a two-valve mouthpiece connected via two lightweight flexible tubes to a computerized oxygen and carbon dioxide gas analysis system [Morgan ventilation monitor (Morgan, Reinham, Kent, UK); oxygen and carbon dioxide analyzers (Ametek SOV S-3A11/Ametek COV CD-3 A, Applied Electrochemistry, Ametek, Pittsburgh, PA)]. The ventilometer and gas analyzers were calibrated before and immediately after each test using a one liter syringe pump and reference gas mixtures, respectively (BOC Gases, Chatswood, Australia). Each treadmill condition was performed until the participant reached a steady state of oxygen consumption (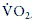) after which a further minute of data was collected for analysis. 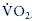 data was collected for a minimum period of four minutes as per the Australian Institute of Sport national testing battery for runners^32^. During the incline conditions, blood lactate concentration levels were determined (Lactate-Pro, Arkray, LT-1710, Kyoto, Japan) after steady state was reached to ensure participants were exercising aerobically (below their previously determined lactate threshold and the 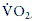 at which this threshold occurred [see Online Supplementary Material]).

In order to compare metabolic and mechanical energy measures, 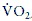 was converted to a metabolic energy cost of transport (J kg^−1^ m^−1^) by using an energy equivalent of 20.1 J ml^−1^ O_2_ and dividing by locomotor speed (m s^−1^) and body mass (kg).

### Arch Compression and 3D Joint Kinematics and Kinetics

**Figure 3:**
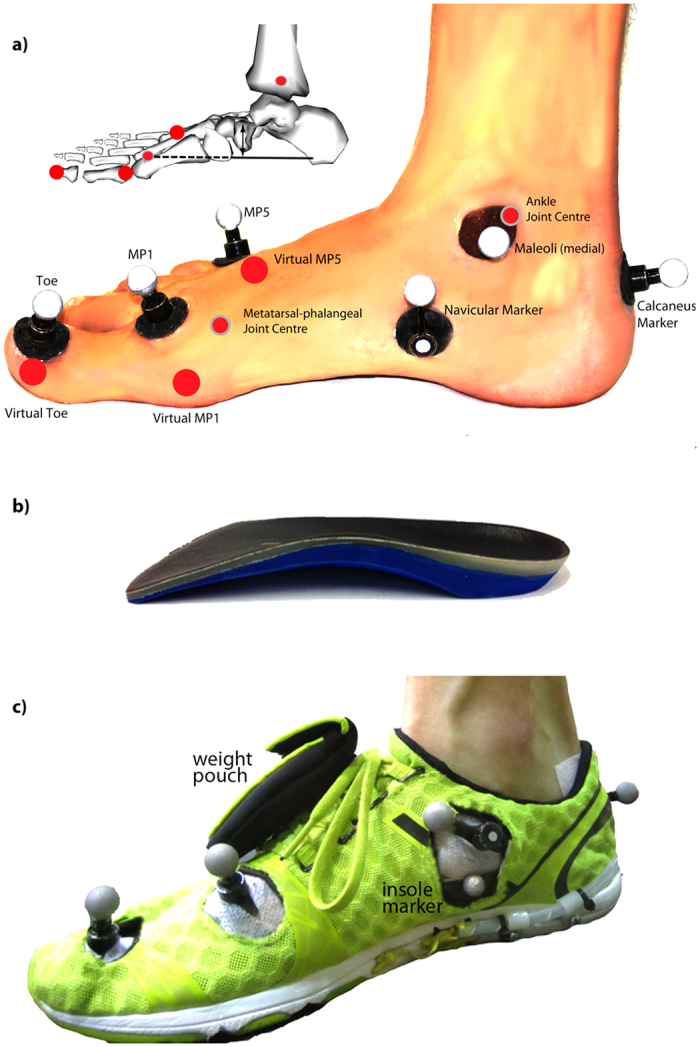
Right foot medial view illustrating (**a**) foot marker positions used to compute foot and arch kinematics. The white markers are physical reflective markers, the large red circles are pointed landmarks and the small red circles represent the computed joint centers. The inset illustrates the virtual landmarks relative to the skeleton, the sole axis and the navicular displacement measure. The inset figure was generated using OpenSim 3.0 (https://simtk.org/home/opensim), freely available open source musculoskeletal modeling software^42^. **(b)** example of the arch compression-restricting insole; image displayed is the Full Arch Insole (FAI); photograph by S.M.S. (**c)** Footwear illustrating marker ‘windows’, the insole marker and weight pouch used for matching total shoe and insole weight; photograph by S.M.S. MP1 = first metatarsophalangeal joint, MP5 = fifth metatarsophalangeal joint.

We used a custom 3D kinematic foot model to estimate arch compression, outlined briefly here, and in detail in the Online Supplementary Material. First, a previously established lower body model was used to define a rearfoot segment and ankle kinematics^33^. Retro-reflective markers were affixed to the lower limbs in accordance with Besier *et al*.^33^, with additional markers placed on the navicular tuberosity and distal phalanx of the first metatarsal (Fig. 3). The shoe upper was modified with five marker ‘windows’ coinciding with the marker positions, allowing markers to be placed directly on the foot and remain visible. To ensure marker positions remained unchanged after removing shoes, all foot markers were detachable by a magnetic base that did not change location. This resulted in the markers being slightly offset from the anatomical landmark. The frame-to-frame location of the marker relative to the anatomical landmark deviated minimally (<1 mm) within their respective rigid segments during running. The location of the functionally relevant anatomical landmarks were identified using a six marker wand in static pointer trials and expressed in the rearfoot or forefoot anatomical coordinate systems (see Online Supplementary Material). A small marker was also placed on the medial aspect of the insole in line with the maximum insole height and hence the maximum arch height.

Motion of the retro-reflective markers were tracked using a ten-camera near infrared Vicon T-series 3D motion capture system (T40S, 250 Hz; Oxford Metrics, Oxford, UK) and sagittal plane high-speed video (300 Hz). Six consecutive right leg strides from the final minute of data collection were selected for analysis. Arch height was defined as the distance of the navicular marker relative to the base of the foot and tracked continuously throughout the stride (see Online Supplementary Material). Arch height at initial foot contact during the minimal shoe-only level run was used as a reference height for each condition. Arch compression was defined as the difference between the reference height and the minimum arch height during stance in each condition.

Inverse dynamics (net joint moments and joint reaction forces) were computed for the ankle in accordance with Besier *et al*.^33^, as well as for the MTP joint (sagittal plane only), using Vicon BodyBuilder software (Oxford Metrics, Oxford, UK). Ground reaction forces (GRFs) from the instrumented treadmill were recorded at 2,000 Hz, and synchronized with the kinematic data using a Vicon MX-Net control box (Oxford Metrics, Oxford, UK). All marker trajectories were filtered using a zero-lag 4^th^ order low pass Butterworth filter with cut-off frequencies typically at 14 Hz, determined by a custom residual analysis algorithm for each participant (MATLAB, The MathWorks Inc., USA). GRFs were filtered at the same cut-off frequency as the kinematic data to mitigate any artefacts in joint moments arising due to un-accounted segment acceleration^34^,^35^.

### Arch Elastic Energy and Total Mechanical Work of Locomotion

**Figure 4:**
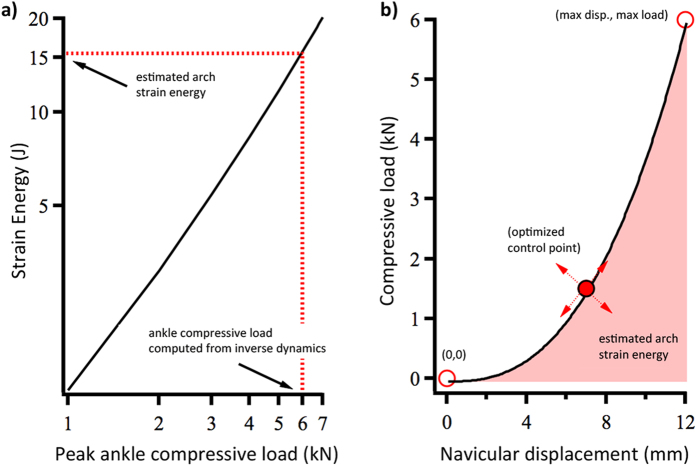
(**a**) Arch elastic strain energy – ankle compressive load relationship adapted from Ker *et al*.^5^ used to estimate arch strain energy from the participant’s maximum ankle joint compressive load (see Online Supplementary Material). (**b**) Subject specific load-displacement curve used to predict stored arch elastic energy during different conditions from measured arch compression. The subject-specific load-displacement curve was established using the maximum arch compression from the participant’s trials and the corresponding ankle compressive load, and by adjusting the optimized control point so that the area under the curve (elastic energy storage) matched the estimated energy storage from (**a**).

Our hypotheses are based on the premise that the insoles impede the storage of elastic energy in the arch and its subsequent ability to contribute to total mechanical work. How these variables changed between conditions was estimated using a simple model to predict arch elastic energy storage (Fig. 4) and a force plate approach to measure total mechanical limb work of locomotion^36^. The arch energy storage model was based on the compressive load-energy storage function established by Ker *et al*.^5^ and calculations of the participants’ individual ankle compressive loads (inverse dynamics) and arch compression (high-speed motion capture) (Fig. 4; see Online Supplementary Material). Using these data, we developed subject-specific arch load-displacement curves that permitted an estimate of the arch elastic energy storage during each trial from their arch compression values (Figs 1a and 4). Arch elastic energy return (*W*_*arch*_^+^) was predicted based on a hysteresis value of 22% from Ker *et al*.^5^. The force plate approach followed the individual limb work (*W*_*limb*_^+^) calculations described by Donelan *et al*.^36^, whereby limb powers were computed as the dot product of the force acting on the limb and the body center of mass velocity, and subsequently integrated with respect to time. Center of mass velocities were computed by time integrating the center of mass accelerations, which were determined from the sum of the ground reaction forces. Both the arch elastic energy and limb work calculations are described in detail in the Online Supplementary Material.

### Modelled Arch Metabolic Energy Saving

The effect of arch elastic energy on the metabolic cost of locomotion was predicted by estimating the amount of positive arch elastic work (energy return) that was eliminated after restricting arch compression, and the cost of replacing lost mechanical work were it to be performed by active muscle. This was computed as:


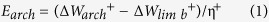


where *E*_*arch*_ is the modelled additional metabolic energy (expressed as a cost of transport; J kg^−1^ m^−1^) after restricting arch compression, ∆*W*_*arch*_^+^ is the difference in the amount of returned arch elastic energy between the minimal shoe-only trial and the corresponding insole trial for level running, incline running and walking (as computed from our model; J kg^−1^ m^−1^). Although they were found to be small (Table 1), we incorporated any differences in positive limb mechanical work between shoe-only and insole conditions (∆*W*_*limb*_^+^) as these could affect metabolic cost. Finally, η^+^ is the muscular efficiency of performing positive work. A constant theoretical muscle efficiency of 0.25 was used for all trials. This assumed that all the lost arch elastic energy was replaced solely by positive muscle fiber work functioning at a high efficiency^37^. For comparison, (presented in the Online Supplementary Material Table S2) we also made predictions using the locomotor mechanical efficiency computed from the minimal shoe-only trial for each condition (walking, level running and incline running).

### Statistics

General linear model two-way repeated measures split-plot ANOVAs were performed to determine the effect of the custom insoles and foot strike technique on the following variables; arch compression, estimated arch elastic energy storage, modeled and experimental metabolic cost, and total limb mechanical work of locomotion. The between subject factors were habitual foot strike technique (RFS and FFS) and the within subjects factors were minimal shoe-only, HAI (level running only) and FAI. The significance level was p < 0.05 for ANOVA analyses. 3 × 2 ANOVAs were conducted for level running and 2 × 2 ANOVAs for walking and incline running. In the 3 × 2 ANOVAs the location of the significant main effect was determined using a *post-hoc* pairwise comparison with a Bonferroni adjustment for multiple comparisons. Cohen’s *d* effect sizes^38^ were calculated and interpreted using the effect scale; small (0.2); moderate (0.5); large (0.8). A series of paired samples t-tests were conducted between RFS and FFS groups in the minimal shoe-only and FAI conditions to determine if foot strike had an effect. Paired samples t-tests were also conducted to compare modelled versus experimentally observed increases in metabolic cost of transport.

## Additional Information

**How to cite this article**: Stearne, S. M. *et al*. The Foot’s Arch and the Energetics of Human Locomotion. *Sci. Rep*. **6**, 19403; doi: 10.1038/srep19403 (2016).

## Supplementary Material

Supplementary Information

Supplementary Video S1

## Figures and Tables

**Table 1:** Arch and limb mechanics and energetics.

Condition	Habitual foot strike	Arch compression (mm)	Estimated elastic energy returned from the arch (J kg^−1^m^−1^)	Observed metabolic cost of transport (J kg^−1^ m^−1^)	Modelled metabolic cost of transport (J kg^−1^ m^−1^)	Total limb mechanical work (J kg^−1^ m^−1^)
WALK						
Minimal shoe-only	RFS	5.9 ± 2.3	0.039 ± 0.032	3.95 ± 0.46	n/a	0.37 ± 0.05
	FFS	7.4 ± 5.3	0.043 ± 0.043	4.06 ± 0.33	n/a	0.42 ± 0.08
	Average	6.7 ± 4.1	0.041 ± 0.037	4.01 ± 0.39	n/a	0.40 ± 0.07
Full Arch Insole (FAI)	RFS	−1.1 ± 5.1	0.004 ± 0.008	3.98 ± 0.50	4.17 ± 0.56	0.39 ± 0.10
	FFS	2.2 ± 4.1	0.003 ± 0.008	4.03 ± 0.42	4.09 ± 0.55	0.38 ± 0.05
	Average	**0.7** ± 4.8*	**0.003** ± 0.007*	4.01 ± 0.44	4.13 ± 0.54	0.39 ± 0.07
LEVEL RUN						
Minimal shoe-only	RFS	**9.2** ± **2.1**^**§**^	0.110 ± 0.034	4.59 ± 0.39	n/a	1.25 ± 0.11
	FFS	**12.0** ± **2.4**^**§**^	0.126 ± 0.055	4.48 ± 0.34	n/a	1.36±0.18
	Average	10.6 ± 2.6	0.118 ± 0.045	4.53 ± 0.36	n/a	1.31±0.15
Half Arch Insole (HAI)	RFS	2.6 ± 4.2	0.017 ± 0.023	4.79 ± 0.38	4.78 ± 0.35	1.20 ± 0.09
	FFS	5.7 ± 6.1	0.021 ± 0.045	4.69 ± 0.42	4.93 ± 0.51	1.36 ± 0.15
	Average	**4.1** ± 5.3*^	**0.019** ± 0.035*	**4.74** ± 0.39*	**4.86** ± 0.43*	1.28 ± 0.15
Full Arch Insole (FAI)	RFS	−1.3 ± 6.1	0.002 ± 0.004	4.87 ± 0.50	4.83 ± 0.48	1.20 ± 0.14
	FFS	2.5 ± 5.8	0.005 ± 0.013	4.74 ± 0.35	4.88 ± 0.50	1.33 ± 0.15
	Average	**0.6** ± 6.0*^	**0.004** ± 0.009*	**4.81** ± 0.42*	**4.85** ± 0.48*	1.27 ± 0.15
INCLINE RUN						
Minimal shoe-only	RFS	9.1 ± 3.4	0.108 ± 0.055	5.88 ± 0.41	n/a	1.46 ± 0.18
	FFS	11.9 ± 4.5	0.116 ± 0.057	5.66 ± 0.26	n/a	1.56 ± 0.17
	Average	10.5 ± 4.1	0.112 ± 0.054	5.77 ± 0.35	n/a	1.51 ± 0.18
Full Arch Insole (FAI)	RFS	−0.3 ± 7.7	0.013 ± 0.028	5.89 ± 0.43	6.04 ± 0.46	1.41 ± 0.19
	FFS	5.4 ± 4.9	0.009 ± 0.016	5.78 ± 0.31	5.93 ± 0.26	1.53 ± 0.10
	Average	**2.5** ± 6.8*	**0.011** ± 0.022*	5.83 ± 0.37	**5.99** ± 0.37*	1.47 ± 0.16

Rearfoot strike (RFS), Forefoot strike (FFS), Average (average of RFS and FFS), Half Arch Insole (HAI), Full Arch Insole (FAI). *Significantly different from minimal shoe-only within the same condition *p* < 0.05, ^§^significant difference between RFS and FFS *p* < 0.05. ^^^Significant difference between HAI and FAI within the same condition *p* < 0.05.

## References

[b1] Cavagna G. A., Heglund N. C. & Taylor C. R. Mechanical work in terrestrial locomotion: Two basic mechanisms for minimizing energy expenditure. Am J Physiol Regulatory Integrative Comp Physiol 233, 243–261 (1977). 10.1152/ajpregu.1977.233.5.R243 411381

[b2] Blickhan R. & Full R. J. In Biomechanics (structures): A practical approach (ed Biewener A.) 75–96 (Oxford University Press, 1992).

[b3] Alexander R. M. Elastic energy stores in running vertebrates. Am Zool 24, 85–94 (1984).

[b4] Rubenson J., Lloyd D. G., Heliams D. B., Besier T. H. & Fournier P. H. Adaptations for economical bipedal running: The effect of limb structure on three-dimensional joint mechanics. J R Soc Inter 8, 740–755, doi: 10.1098/​rsif.2010.0466 (2011). PMC3061092 21030429

[b5] Ker R. F., Bennett M. B., Bibby S. R., Kester R. C. & Alexander R. M. The spring in the arch of the human foot. Nature 325, 147–149 (1987). 3808070 10.1038/325147a0

[b6] Perl D., Daoud A. & Lieberman D. Effects of footwear and strike type on running economy. Med Sci Sports Exerc 44, 1335–1343, doi: 10.1249/MSS.0b013e318247989e (2012). 22217565

[b7] Bramble D. M. & Lieberman D. E. Endurance running and the evolution of *homo*. Nature 432, 345–352, doi: 10.1038/nature03052 (2004). 15549097

[b8] Morgan D. W., Miller T. A., Mitchell V. A. & Craib M. W. Aerobic demand of running shoes designed to exploit energy storage and return. Res Q Exercise Sport 67, 102–105, doi: 10.1080/02701367.1996.10607931 (1996). 8736000

[b9] Nilsson J. & Thorstensson A. Ground reaction forces at different speeds of human walking and running. Acta Physiol Scand 136, 217–227, doi: 10.1111/j.1748-1716.1989.tb08655.x (1989). 2782094

[b10] Hicks J. The mechanics of the foot: Ii. The plantar aponeurosis and the arch. J Anat 88, 2–30 (1954). PMC1244640 13129168

[b11] Snyder K. L., Kram R. & Gottschall J. S. The role of elastic energy storage and recovery in downhill and uphill running. J Exp Biol 215, 2283–2287, doi: 10.1242/jeb.066332 (2012). 22675189

[b12] Fukunaga T., Kawakami Y., Kubo K. & Kanehisa H. Muscle and tendon interaction during human movements. Exerc Sport Sci Rev 30, 106–110, doi: 0091-6631/3003/106–110 (2002). 12150568 10.1097/00003677-200207000-00003

[b13] Fletcher J. R. & MacIntosh B. R. Achilles tendon strain energy in distance running: Consider the muscle energy cost. J Appl Physiol 118, 193–199, doi: 10.1152/japplphysiol.00732.2014 (2015). 25593218 PMC4297774

[b14] Otman S., Basgoze O. & Gokce-Kutsal Y. Energy cost of walking with flat feet. Prosthey Orthot Int 12, 73–76 (1988). 10.3109/03093648809078203 3174409

[b15] Karimi M. T., Fereshtehnejad N. & Pool F. The impact of foot insole on the energy consumption of flat-footed individuals during walking. Foot Ankle Spec 6, 21–26, doi: 10.1177/1938640012457676 (2013). 22956661

[b16] Berg K. & Sady S. Oxygen cost of running at submaximal speeds while wearing shoe inserts. Res Q Exercise Sport 56, 86–89, doi: 10.1080/02701367.1985.10608438 (1985).

[b17] Burkett L. N., Kohrt W. M. & Buchbinder R. Effects of shoe and foot orthotics on vo2 and selected frontal plane knee kinematics. Med Sci Sports Exerc 17, 158–163 (1985). 3982270

[b18] Hayes P. & Caplan N. Foot strike patterns and ground contact times during high-calibre middle-distance races. J Sport Sci 30, 1275–1283, doi: 10.1080/02640414.2012.707326 (2012). 22857152

[b19] Frederick E. C. Physiological and ergonomics factors in running shoe design. Appl Ergon 15, 281-287, doi: 0003-6870/84/04 0281–07 (1984). 10.1016/0003-6870(84)90199-6 15676526

[b20] White T. D. & Suwa G. Hominid footprints at laetoli: Facts and interpretations. Am J Phys Anthropol 72, 485–514, doi: 10.1002/ajpa.1330720409 (1987). 3111270

[b21] Harcourt-Smith W. E. H. & Aiello L. C. Fossils, feet and the evolution of human bipedal locomotion. J Anat 204, 403–416, doi: 10.1111/j.0021-8782.2004.00296.x (2004). 15198703 PMC1571304

[b22] Ward C. V., Kimbel W. H. & Johanson D. C. Complete fourth metatarsal and arches in the foot of australopithecus afarensis. Science 311, 750-753, doi: 0.1126/science.1201463 (2011). 21311018 10.1126/science.1201463

[b23] Lieberman D. E. Human evolution: Those feet in ancient times. Nature 483, 550–551 (2012). 22460898 10.1038/483550a

[b24] Morton D. J. Evolution of the longitudinal arch of the human foot. J Bone Joint Surg Am 6, 56–90 (1924).

[b25] Bojsen-Møller F. Calcaneocuboid joint and stability of the longitudinal arch of the foot at high and low gear push off. J Anat 129, 165–176 (1979). 511760 PMC1233091

[b26] Elftman H. & Manter J. Chimpanzee and human feet in bipedal walking. Am J Phys Anthropol 20, 69–79, doi: 10.1002/ajpa.1330200109 (1935).

[b27] Redmond A. C., Crosbie J. & Ouvrier R. A. Development and validation of a novel rating system for scoring standing foot posture: The foot posture index. Clin Biomech 21, 89–98, doi: 10.1016/j.clinbiomech.2005.08.002 (2006). 16182419

[b28] Tung K. D., Franz J. R. & Kram R. A test of the metabolic cost of cushioning hypothesis during unshod and shod running. Med Sci Sports Exerc 46, 324–329, doi: 10.1249/MSS.0b013e3182a63b81 (2014). 24441213

[b29] Cavanagh P. R. & Lafortune M. A. Ground reaction forces in distance running. J Biomech 13, 397-406, doi: 0021-9290/80/0501-0397 S02.00/0 (1980). 10.1016/0021-9290(80)90033-0 7400169

[b30] Lieberman D. . Foot strike patterns and collision forces in habitually barefoot versus shod runners. Nature 463, 531–535, doi: 10.1038/nature08723 (2010). 20111000

[b31] Minetti A. E., Ardigò L. P. & Saibene F. Mechanical determinants of the minimum energy cost of gradient running in humans. J Exp Biol 195, 211–225 (1994). 7964412 10.1242/jeb.195.1.211

[b32] Saunders P. U. & Green D. J. In Physiological tests for elite athletes 2nd ed. (eds Tanner R. K. & Gore C. J.) 397–409 (Human Kinetics, 2013).

[b33] Besier T. F., Sturnieks D. L., Alderson J. A. & Lloyd D. G. Repeatability of gait data using a functional hip joint centre and a mean helical knee axis. J Biomech 36, 1159–1168, doi: 10.1016/S0021-9290(03)00087-3 (2003). 12831742

[b34] Bisseling R. W. & Hof A. L. Handling of impact forces in inverse dynamics. J Biomech 39, 2438–2444, doi: 10.1016/j.jbiomech.2005.07.021 (2006). 16209869

[b35] Kristianslund E., Krosshaug T. & van den Bogert A. Effect of low pass filtering on joint moments from inverse dynamics: Implications for injury prevention. J Biomech 45, 666–671, doi: 10.1016/j.jbiomech.2011.12.011 (2012). 22227316

[b36] Donelan M. J., Kram R. & Kuo A. D. Simultaneous positive and negative external mechanical work in human walking. J Biomech 35, 117–124, doi: 10.1016/S0021-9290(01)00169-5 (2002). 11747890

[b37] Woledge R. C., Curtin N. A. & Homsher E. Energetic aspects of muscle contraction. Monogr Physiol Soc 41, 1–357 (1985). 3843415

[b38] Cohen J. Statistical power analysis for the behavioral sciences. 2nd edition edn, (Lawrence Erlbaum Associates Publishers, 1988).

