# Supplementary material for: The Foot’s Arch and the Energetics of Human Locomotion
Source: Sci Rep. 2016 Jan 19;6:19403. doi: 10.1038/srep19403 (PMC4726102; doi:10.1038/srep19403)
Supplement: Supplementary Information [file srep19403-s1.pdf]

**The Foot's Arch and the Energetics of Human Locomotion**

Sarah M. Stearne[1], Kirsty A. McDonald[1], Jacqueline A. Alderson[1], Ian North[2], Charles E. Oxnard[3] &

*Jonas Rubenson[1,4]

[1] School of Sport Science, Exercise and Health, The University of Western Australia, Perth, WA, 6009, Australia

[2] Willetton Podiatry, Willetton, WA, 6155, Australia

[3] Anatomy, Physiology and Human Biology, The University of Western Australia, Perth, WA, 6009, Australia

[4]Biomechanics Laboratory, Department of Kinesiology, The Pennsylvania State University, University Park, PA, 16803, USA

# Online Supplementary Material

## Additional Methodology

*Insole Material Testing*

To assess the energy storage/return of the insoles themselves during running the material properties of the insoles were tested using an Instron material testing machine with a custom testing mount (Instron Model 8874, Illinois Tool Works Inc.) and Instron Dynamic Software (Wave Matrix Version 1.2). A custom Acetal plastic testing mount (diameter 50 mm) that conformed to the slope and curve of the insole arch (point of max height) was designed. Force was applied to the insoles in a manner that replicated measured RFS and FFS stance time and stride frequency [1]. The Instron was programmed to displace the insoles four millimeters during running for 100 cycles and the resultant load displacement was recorded allowing measurement of the insole stiffness and energy storage/return (Supplementary Figure S1). The value of compression was chosen to encompass the maximum insole compression measured during pilot testing of 2.7 $ms^{-1}$ running (measured with a high speed camera). For a video of the insole material testing please see additional Online Supplementary Material.

**Supplementary Video S1.** High-speed video (300 frames $s^{-1}$) of the insole material testing. The material testing rig (Instron Model 8874, Illinois Tool Works Inc.) was configured to compress the insole 4mm during a simulated running foot contact).

*Foot Model and Arch Compression Estimates*

Because the markers placed on the first and fifth metatarsal bases were elevated from the foot, we used static pointer trials to identify the anatomical medial and lateral aspects of these landmarks, expressed in the rearfoot anatomical coordinate system. The midpoint of these virtual landmarks was used to define both a rearfoot-forefoot joint center (metatarsophalangeal (MTP) joint center) as well as the base of the foot (Manuscript Figure 3). A forefoot segment was created using the virtual metatarsal markers and a third virtual hallux marker, defined using a static pointer trial (these virtual landmarks were expressed relative to a coordinate system defined by the metatarsal and hallux

markers). A foot sole plane was created by transposing the rearfoot coordinate system so that its origin was the rearfoot-forefoot joint center (the x-z-plane of this coordinate system is oriented parallel with the ground in a neutral standing posture). A continuous trace of the vertical displacement of the navicular marker relative to the new rearfoot coordinate system in the y-axis was used as a measure of arch compression (Supplementary Figure S2). This method minimized any effects that ankle inversion/eversion has on predicting arch compression since these motions do not alter the location of the navicular marker in the rearfoot coordinate system. The model also allowed us to compute the inversion/eversion angle, which was found to be unaffected by the insole (Supplementary Figure S3). The navicular height relative to the sole of the foot (x-z-plane of the rear-foot coordinate system) at initial foot contact in the shod only condition was used as a reference value to standardize arch compression. Results were compared to digitized high speed video footage (navicular marker relative to top of the shoe midsole); maximal navicular compression from the two methods correlated strongly (r = 0.88).

Sagittal plane joint angles and net moments of the forefoot segment were calculated about the MTP joint center using Vicon BodyBuilder software (Oxford Metrics, Oxford, UK). The ground reaction force (GRF) was ascribed to the forefoot segment when the center of pressure was anterior to the MTP joint center and to the rearfoot when it was distal. Inverse dynamic calculations of the forefoot segment assumed that the moments generated by the mass and inertia of the segment were negligible as per Stefanyshyn and Nigg [2].

*Arch Elastic Energy and Total Limb Mechanical Work of Locomotion Estimates*

First, the maximal experimental ankle compressive load during stance was estimated by summing the computed joint reaction force with an estimate of the Achilles tendon force (the trial with the highest value was used). Achilles tendon force was estimated by dividing the net ankle joint moment by the Achilles tendon moment arm taken from calliper measurements during standing. The

participant's peak arch strain energy was then predicted from the estimated compressive ankle load using the strain energy versus ankle load relationship from Ker et al. [3] (their Figure 2B was digitized and used to develop the strain energy equation (J) = $0.269x^2 + 1.004x$; where x = ankle compressive load (kN)). We subsequently developed subject specific arch load-displacement curves (see Manuscript Figure 4) using two fixed points, (0,0) and (max load, max arch compression), and varied a third point so that the area under the line of best fit (power function) for the three load-displacement points matched the energy storage predicted from the above strain energy equation (power function; $F_{comp} = y_0 + Ax^{pow}$, where $F_{comp}$ is the compressive force (kN), $y_0$ is the *y*-intercept (0), *x* is arch compression (mm) and *A* is a constant). The optimization procedure was performed using a root solver algorithm in MATLAB (fsolve; The MathWorks, Natick, MA). The subject-specific arch load-displacement curve was subsequently used to estimate the elastic energy stored under other amounts of arch compression in the remaining walking/running trials by integrating the area under the arch load vs displacement curve for the specified arch compression. The amount of energy returned to the runner from the estimated stored arch elastic energy ($W_{arch}^{+}$) was calculated using a hysteresis of 22%, based on data from Ker et al. [3]. The $W_{arch}^{+}$ was expressed in J $kg^{-1}$ $m^{-1}$ by multiplying the returned energy from the left foot by two (assuming symmetry) and dividing by the average distance traveled per stride and body mass.

The positive mechanical work during a single step ($W_{limb,step}^{+}$) was calculated for walking, level running and incline running using the individual limb work force plate approach described by Donelan et al.[4]. Limb powers for the right and left limbs ($P_r$, $P_l$) were calculated (from right heel strike to left heel strike) as the dot product of the ground reaction force acting on the right and left limbs ($F_r$, $F_l$), respectively, and the velocity of the center of mass ($v_{COM}$) (Eq. S1). The right and left limb ground reaction forces were recorded from independent force plates under the right and left treadmill belts.

$$P_r = \vec{F}_r \cdot \vec{v}_{COM} = F_{z_r} v_{z,COM} + F_{y_r} v_{y,COM} + F_{x_r} v_{x,COM} \qquad \text{(Eq. S1)}$$

$$P_l = \vec{F}_l \cdot \vec{v}_{COM} = F_{z_l} v_{z,COM} + F_{y_l} v_{y,COM} + F_{x_l} v_{x,COM}$$

Centre of mass (COM) velocities (vertical, z; fore-aft, y; medio-lateral, x) were initially calculated by time-integrating the COM accelerations, determined from the sum of the right and left limb GRF's (Eq. S2) (see Donelan [4]).

$$v_{z,COM} = \int \frac{F_{z_r} + F_{z_l} - mg}{m} dt \qquad \text{(Eq. S2)}$$

$$v_{y,COM} = \int \frac{F_{y_r} + F_{y_l}}{m} dt$$

$$v_{x,COM} = \int \frac{F_{x_r} + F_{x_l}}{m} dt$$

where *m* is body mass and *g* is acceleration due to gravity (9.81 m $s^{-1}$). During a single running step the GRF from the trailing limb (designated to be the left limb) equaled zero. Integration constants (offsets) were applied to the calculated COM velocities: z was set so that the average COM vertical velocity over one step equaled 0; y was set so that the average fore-aft velocity over one step equaled the treadmill speed; x was set so that the medio-lateral velocity at the start and end of the step were equal but opposite. Offsets were adjusted during the incline condition: z; treadmill speed x sin 3°, y; treadmill speed x cos 3°. Limb powers were restricted to positive values and integrated with respect to time ($t_i$ and $t_f$ represent the right heel strike and left heel strike respectively) to determine the positive mechanical work performed in the limbs over one step (Eq. S3).

$$W^{+}_{right} = \int_{t_i}^{t_f} P_r dt, \text{ for } P_r > 0 \qquad \text{(Eq. S3)}$$

$$W^{+}_{left} = \int_{t_i}^{t_f} P_l dt, \text{ for } P_l > 0$$

where $W^{+}_{right}$ and $W^{+}_{left}$ represent the positive mechanical work of the right and left limb, respectively. To compute the total positive mechanical work during the step ($W^{+}_{limb,step}$) in walking, $W^{+}_{right}$ and $W^{+}_{left}$ were summed as per the individual limbs methods described by Donelan et al. [4]. In running, the left (trailing) limb generated no ground reaction force as it was in swing-phase and thus $W^{+}_{limb,step}$ was taken as $W^{+}_{right}$. The mechanical cost of transport ($W^{+}_{limb}$; J kg$^{-1}$ m$^{-1}$) was computed by multiplying $W^{+}_{limb,step}$ by two (assuming bilateral limb symmetry) and dividing by the average distance traveled per stride and body mass.

*Locomotor Mechanical Efficiency and Modelled Metabolic Energy Prediction*

In addition to predicting increase in metabolic cost using a constant efficiency of 25%, we also predicted costs using a computed mechanical locomotor efficiency ($\eta^{+}_{loc}$). This was done by using the experimental gross energy cost ($E_{tot}$; J kg$^{-1}$ m$^{-1}$) and the positive limb mechanical work ($W_{limb}^{+}$; J kg$^{-1}$ m$^{-1}$) of the minimal shoe-only conditions:

$$\eta^{+}_{loc} = W_{limb}^{+} / E_{tot} \qquad \text{(Eq. S4)}$$

We subsequently computed a predicted increase in locomotor metabolic cost associated with restricting arch compression for each condition as:

$$E_{arch} = (\Delta W_{arch}^{+} - \Delta W_{limb}^{+}) \,/\, \eta^{+}_{loc} \qquad \text{(Eq. S5)}$$

where $\Delta W_{arch}^{+}$ and $\Delta W_{limb}^{+}$ are the differences in the amount of returned arch elastic energy and positive limb mechanical work between the minimal shoe-only trial and the corresponding insole trial for level running, incline running and walking, respectively.

*Maximal Oxygen Consumption and Lactate Threshold*

In a separate session prior to the biomechanics testing, participant's maximum oxygen uptake ($\dot{V}O_{2max}$) and lactate threshold were determined by an incremental exercise test. In the 24 hours prior to attending the laboratory, participants were asked not to exercise heavily or consume caffeinated food or beverages. In the two hours prior participant were instructed not to consume any food or drink other than water. Before commencement of the test, a baseline heart rate (Polar F1 Heart Rate Monitor, Kempele, Finland) and fingertip capillary blood sample (Lactate-Pro, Arkray, LT-1710, Kyoto, Japan) were collected. Participants were allowed to warm up at a self-selected pace and familiarize themselves with the motorized treadmill (VR 3000, NuryTech Inc, Germany) and mouth piece.

A three minute exercise and one minute rest protocol was followed until volitional exhaustion. Given the weekly running distance inclusion criteria of the study, it was assumed that all participants were of a good fitness level so all $\dot{V}O_{2max}$ tests commenced at a standard 10 km/hr. The treadmill belt speed was increased by 2 km/hr after each exercise bout until 16 km/hr after which speed was increased by 1 km/hr. During the rest minute participants straddled the treadmill belt while a fingertip blood sample was collected and heart rate recorded. Throughout the test, expired gasses were collected by a two-valve mouthpiece connected via two lightweight flexible tubes to a computerized oxygen and carbon dioxide gas analysis system [oxygen and carbon dioxide analysers (Ametek SOV S-3A11 / Ametek COV CD-3A, Applied Electrochemistry, Ametek, Pittsburgh, PA)]. Ventilation was recorded at 15 second intervals using a turbine ventilometer (225A; Morgan, Chatham Kent, UK). The ventilometer and gas analysers were calibrated before and immediately after each test using a one litre syringe pump and reference gas mixtures, respectively (BOC Gases, Chatswood, Australia). Participant's peak oxygen consumption was determined by summing the four highest consecutive 15 second $VO_2$ values. The lactate threshold was determined using a Dmax method [5].

*Additional Statistical Analysis*

A series of Wilcoxon's Signed Rank Tests (non-parametric) were used to assess statistical differences between RPE and questionnaire results during walking, level running and incline running conditions.

## Additional Results

*Temporal Parameters*

The insoles had no effect on any temporal parameters (stance, swing or stride time). Foot strike technique significantly affected stance time ($p$ = 0.014) in level running and on both stance ($p$ = 0.019) and swing time ($p$ = 0.040) in incline running. RFS runners spent longer in the stance phase in both conditions and a shorter time in the swing phase during the incline condition compared with FFS runners. See Table S1 for additional temporal parameter data.

*Incremental Exercise Test*

The incremental exercise test identified that participant's lactate threshold was 6.1 ± 1.1 mmol/L which occurred at a speed of 4.5 ± 0.3 $ms^{-1}$. Given that the energy expenditure and lactate concentrations when running at 2.7 $ms^{-1}$ on both the level and incline were lower than that at which the lactate threshold occurred, (lactate concentrations; incline running shoe-only 2.5 ± 1.0 mmol/L, incline running FAI 2.5 ± 1.2 mmol/L), it was concluded that all participants were exercising aerobically. The blood lactate concentration of one participant during the incline trial exceeded his previously determined threshold. His incline and level running data were therefore removed from the analyses.

*Insole Material Testing*

Material testing on the insoles returned less than 0.4 Joules of energy during running (Figure S1). This value is equal to less than 3% of the elastic energy storage/return that was estimated to be lost when wearing the insoles.

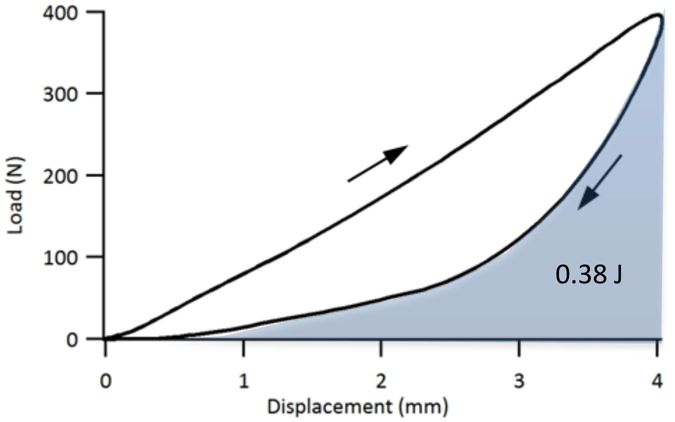


**Supplementary Figure S1.** Average insole load-displacement curve indicating minimal energy return from the insoles.

*Arch Kinematics*

A representative trace of arch compression over the stance phase of level running for one participant is presented in Figure S2. Peak arch compression was significantly reduced in the HAI and FAI conditions compared to the minimal shoe-only (both $p < 0.001$; Figure S2). No statistically significant difference in peak ankle inversion or eversion was identified between the minimal shoe-only and FAI level running conditions (Figure S3).

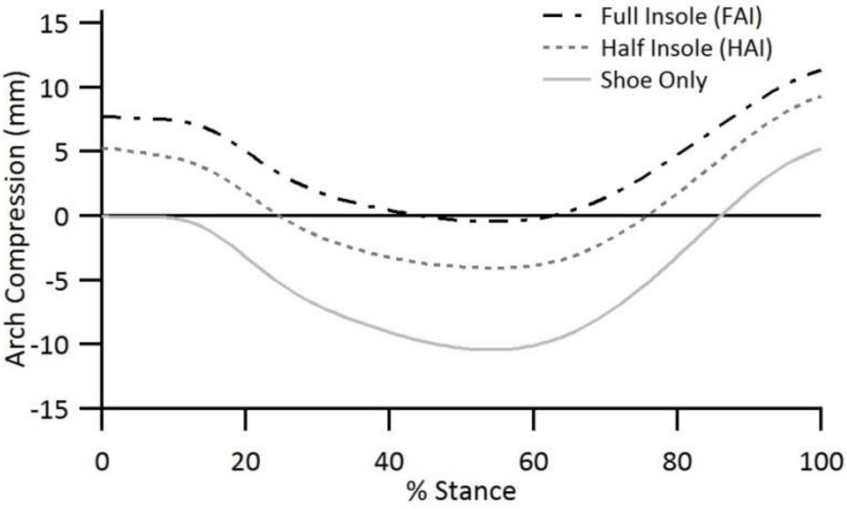


**Supplementary Figure S2.** Representative arch compression (mm) data when running in the minimal shoe only (solid light grey line), half arch insole (HAI; grey dashed line) and full arch insole (FAI; black dashed line) throughout the stance phase in level running. Zero arch compression indicates arch height at initial foot contact in the minimal shoe-only condition. Positive values indicate a slackened state of the arch elastic structures compared to the initial foot contact in the minimal shoe only condition and negative values indicate stretch compared to the minimal shoe only initial foot contact.

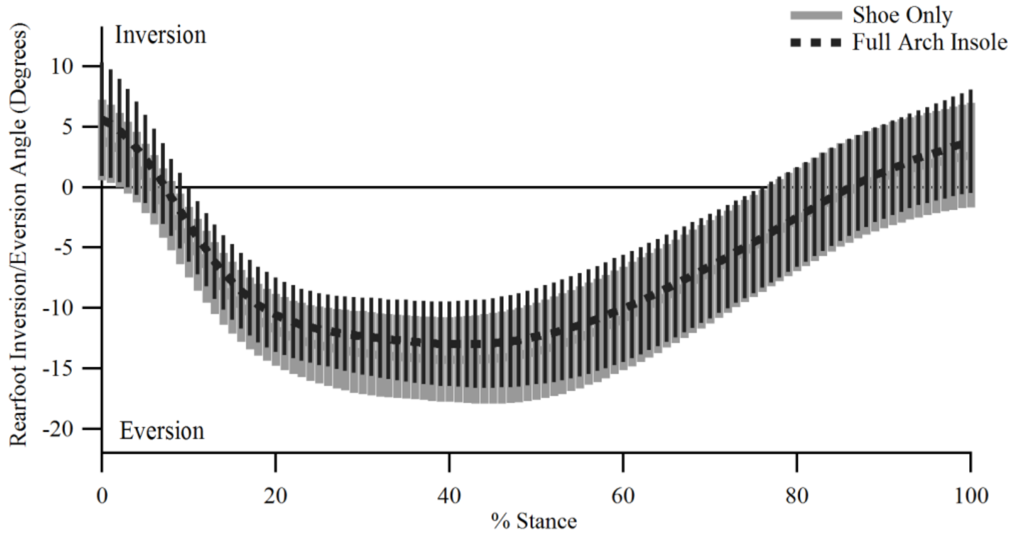


**Supplementary Figure S3.** Average rearfoot inversion/eversion angle (± S.D.) across the stance phase of level running (from foot contact to toe off) during the shoe only (grey) and Full Arch Insole (FAI; black) conditions. No statistically significant ($p < 0.05$) difference was observed between initial, peak, or terminal stance eversion angles.

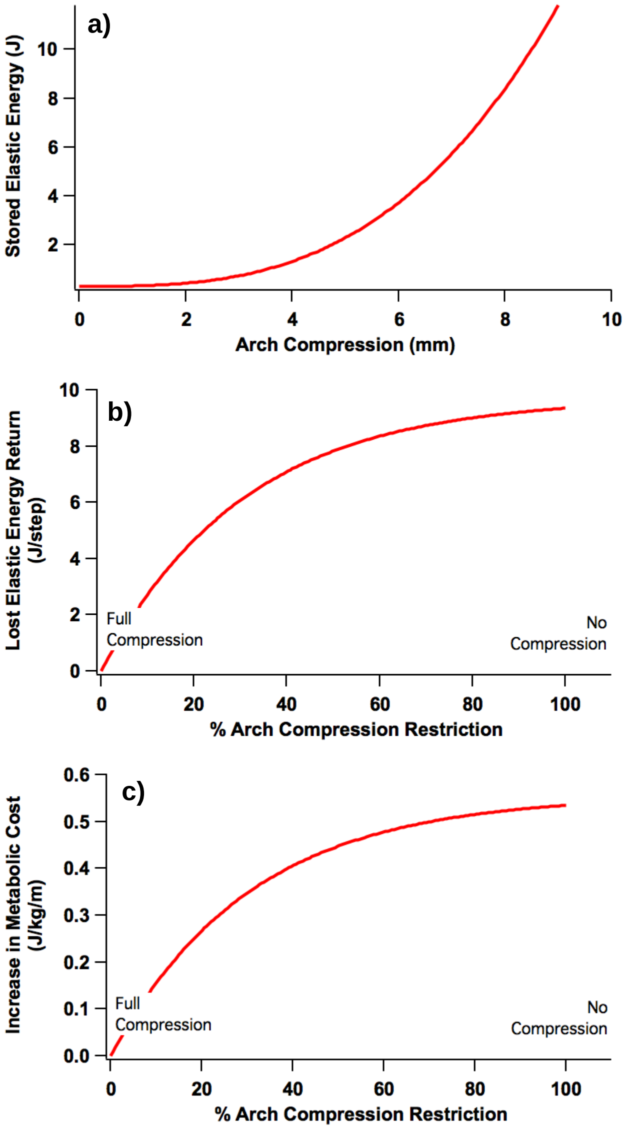


**Supplementary Figure S4.** Typical participant arch mechanics and energetics predicted from our model, indicating; **a)** the stored elastic energy vs arch compression, **b)** the predicted energy lost in the arch spring as a function of the % arch compression restriction and **c)** the predicted increase in the metabolic energy cost of running (J $kg^{-1}$ $m^{-1}$; level running at 2.7 $ms^{-1}$) as a function of the % arch compression restriction.

**Supplementary Table S1.** Temporal parameters during walking, level running and incline running across shoe only, half arch insole (HAI) and full ach insole (FAI) in rearfoot strike (RFS) and forefoot strike (FFS) groups.

| | | walk | | | level run | | | incline run | | |
|---|---|---|---|---|---|---|---|---|---|---|
| | | **stance time** | **swing time** | **stride time** | **stance time** | **swing time** | **stride time** | **stance time** | **swing time** | **stride time** |
| main Effect *p value* | insole | *0.142* | *0.896* | *0.151* | *0.551* | *0.126* | *0.197* | *0.650* | *0.797* | *0.374* |
| | foot strike | *0.144* | *0.427* | *0.624* | ***0.014*†** | *0.079* | *0.512* | ***0.01**9*† | ***0.040*†** | *0.798* |
| **shoe only** | rearfoot strike (RFS) | 0.77 ± 0.03 | 0.45 ± 0.02 | 1.22 ± 0.05 | **0.30 ± 0.03*** | 0.44 ± 0.05 | 0.74 ± 0.05 | **0.31 ± 0.04*** | **0.43 ± 0.06*** | 0.73 ± 0.05 |
| | forefoot strike (FFS) | 0.74 ± 0.03 | 0.45 ± 0.02 | 1.19 ± 0.05 | **0.28 ± 0.03*** | 0.46 ± 0.05 | 0.73 ± 0.05 | **0.26 ± 0.01*** | **0.48 ± 0.05*** | 0.74 ± 0.05 |
| **half arch insole (HAI)** | rearfoot strike (RFS) | | | | **0.30 ± 0.03*** | 0.43 ± 0.05 | 0.73 ± 0.05 | | | |
| | forefoot strike (FFS) | | | | **0.26 ± 0.01*** | 0.49 ± 0.06 | 0.75 ± 0.06 | | | |
| **full arch insole (FAI)** | rearfoot strike (RFS) | 0.78 ± 0.04 | 0.45 ± 0.04 | 1.23 ± 0.07 | **0.30 ± 0.03*** | 0.44 ± 0.05 | 0.74 ± 0.05 | **0.29 ± 0.03*** | **0.44 ± 0.05*** | 0.73 ± 0.04 |
| | forefoot strike (FFS) | 0.76 ± 0.04 | 0.44 ± 0.02 | 1.20 ± 0.05 | **0.26 ± 0.02*** | 0.47 ± 0.07 | 0.73 ± 0.07 | **0.27 ± 0.02*** | **0.46 ± 0.07*** | 0.74 ± 0.06 |

(All times are in seconds. † indicates significant ANOVA main effect ($p < 0.05$). Where a significant main effect was detected, t-tests were run to determine the location of the effect. * indicates significant t-test difference ($p < 0.05$) between habitual rearfoot strike (RFS) and habitual forefoot strike (FFS) runners within condition.)

**Supplementary Table S2.** Additional data on arch compression restriction during walking, level running and incline running by the Half (HAI; level run only) and Full Arch Insoles (FAI) and observed and modelled metabolic changes.

| Condition | Habitual foot strike | % Arch compression restricted | Net $\dot{V}O_2$ (minus standing $\dot{V}O_2$) (ml kg$^{-1}$ min$^{-1}$) | Gross $\dot{V}O_2$ (ml kg$^{-1}$ min$^{-1}$) | Experimentally observed change in metabolic cost of transport (gross $\dot{V}O_2$) (J kg$^{-1}$ m$^{-1}$) | Experimentally observed % change in metabolic cost of transport (vs shoe-only) | Modelled change in metabolic cost of transport (25% efficiency) (J kg$^{-1}$ m$^{-1}$) | Modelled % change in metabolic cost of transport (25% efficiency) (vs shoe-only) | Calculated locomotor efficiency | Modelled change in metabolic cost of transport (calculated locomotor efficiency) (J kg$^{-1}$ m$^{-1}$) | Modelled % change in metabolic cost of transport (calculated locomotor efficiency) (vs shoe-only) |
|---|---|---|---|---|---|---|---|---|---|---|---|
| WALK | | | | | | | | | | | |
| Minimal shoe-only | RFS | n/a | 6.3 ± 1.2 | 13.0 ± 1.5 | n/a | n/a | n/a | n/a | 9.5 ± 1.4 | n/a | n/a |
| | FFS | n/a | 6.6 ± 0.8 | 13.3 ± 1.1 | n/a | n/a | n/a | n/a | 10.5 ± 2.4 | n/a | n/a |
| | Average | n/a | 6.5 ± 1.0 | 13.2 ± 1.3 | n/a | n/a | n/a | n/a | 10.0 ± 2.0 | n/a | n/a |
| Full Arch Insole (FAI) | RFS | 84.6 ± 22.4 | 6.4 ± 1.1 | 13.1 ± 1.6 | 0.04 ± 0.38 | 1.3 ± 10.3 | 0.22 ± 0.27 | 5.7 ± 6.3 | n/a | 0.58 ± 0.75 | 14.5 ± 17.5 |
| | FFS | 80.5 ± 21.1 | 6.5 ± 0.6 | 13.2 ± 1.4 | -0.01 ± 0.25 | -0.6 ± 6.3 | 0.03 ± 0.33 | 0.6 ± 7.8 | n/a | 0.22 ± 0.96 | 4.8 ± 20.9 |
| | Average | **82.4 ± 21.1*** | 6.5 ± 0.8 | 13.2 ± 1.5 | 0.02 ± 0.31 | 0.3 ± 8.2 | 0.12 ± 0.31 | 3.0 ± 7.4 | n/a | 0.39 ± 0.83 | 9.4 ± 19.4 |
| LEVEL RUN | | | | | | | | | | | |
| Minimal shoe-only | RFS | n/a | 30.3 ± 2.7 | 37.0 ± 3.1 | n/a | n/a | n/a | n/a | 27.5 ± 3.4 | n/a | n/a |
| | FFS | n/a | 29.4 ± 2.5 | 36.1 ± 2.7 | n/a | n/a | n/a | n/a | 30.3 ± 3.4 | n/a | n/a |
| | Average | n/a | 29.9 ± 2.5 | 36.5 ± 2.9 | n/a | n/a | n/a | n/a | 28.9 ± 3.6 | n/a | n/a |
| Half Arch Insole (HAI) | RFS | 63.5 ± 33.0 | 31.9 ± 2.7 | 38.6 ± 3.1 | 0.20 ± 0.20 | 4.5 ± 4.6 | 0.20 ± 0.18 | 4.5 ± 4.0 | n/a | 0.18 ± 0.17 | 4.0 ± 3.6 |
| | FFS | 59.3 ± 35.8 | 31.0 ± 3.3 | 37.8 ± 3.4 | 0.20 ± 0.26 | 4.5 ± 5.8 | 0.45 ± 0.31 | 10.0 ± 6.9 | n/a | 0.39 ± 0.30 | 8.6 ± 6.4 |
| | Average | **61.4 ± 33.3*** | **31.5 ± 3.0*** | **38.2 ± 3.2*** | **0.20 ± 0.23*** | **4.5 ± 5.0*** | **0.32 ± 0.28*** | **7.2 ± 6.1*** | n/a | **0.28 ± 0.26*** | **6.3 ± 5.6*** |
| Full Arch Insole (FAI) | RFS | 84.7 ± 22.0 | 32.6 ± 3.5 | 39.3 ± 4.0 | 0.28 ± 0.23 | 6.2 ± 4.7 | 0.24 ± 0.30 | 5.3 ± 6.2 | n/a | 0.21 ± 0.28 | 4.7 ± 5.9 |
| | FFS | 73.0 ± 27.3 | 31.5 ± 2.6 | 38.2 ± 2.8 | 0.26 ± 0.17 | 5.9 ± 3.8 | 0.40 ± 0.34 | 8.9 ± 7.8 | n/a | 0.34 ± 0.31 | 7.6 ± 7.0 |
| | Average | **78.9 ± 24.7*** | **32.1 ± 3.1*** | **38.7 ± 3.4*** | **0.27 ± 0.20*** | **6.0 ± 4.2*** | **0.32 ± 0.32*** | **7.1 ± 7.1*** | n/a | **0.28 ± 0.30*** | **6.2 ± 6.4*** |
| INCLINE RUN | | | | | | | | | | | |
| Minimal shoe-only | RFS | n/a | 40.7 ± 2.7 | 47.4 ± 3.3 | n/a | n/a | n/a | n/a | 24.9 ± 3.4 | n/a | n/a |
| | FFS | n/a | 38.9 ± 2.2 | 45.6 ± 2.1 | n/a | n/a | n/a | n/a | 27.5 ± 2.3 | n/a | n/a |
| | Average | n/a | 39.8 ± 2.6 | 46.5 ± 2.8 | n/a | n/a | n/a | n/a | 26.2 ± 3.1 | n/a | n/a |
| Full Arch Insole (FAI) | RFS | 76.2 ± 33.2 | 40.8 ± 2.8 | 47.5 ± 3.5 | 0.01 ± 0.09 | 0.2 ± 1.5 | 0.16 ± 0.37 | 2.8 ± 6.4 | n/a | 0.14 ± 0.35 | 2.5 ± 6.0 |
| | FFS | 60.8 ± 27.6 | 39.8 ± 2.7 | 46.6 ± 2.5 | 0.12 ± 0.24 | 2.1 ± 4.2 | 0.28 ± 0.33 | 5.1 ± 6.0 | n/a | 0.27 ± 0.33 | 4.9 ± 6.0 |
| | Average | **68.5 ± 30.6*** | 40.3 ± 2.7 | 47.0 ± 3.0 | 0.07 ± 0.18 | 1.2 ± 3.2 | **0.22 ± 0.34*** | **4.0 ± 6.1*** | n/a | **0.20 ± 0.33*** | **3.7 ± 5.9*** |

Rearfoot strike (RFS), Forefoot strike (FFS), Average (average of RFS and FFS), Half Arch Insole (HAI), Full Arch Insole (FAI). All statistical analyses were conducted on raw data not percentages. ANOVAs revealed no main effect of foot strike. * Significantly different from minimal shoe-only within the same condition $p < 0.05$.

**Supplementary Table S3.** Questionnaire results.

| Question | Group | Walk | Level Run | | Incline Run |
|---|---|---|---|---|---|
| | | full arch insole (FAI) | half arch insole (HAI) | full arch insole (FAI) | full arch insole (FAI) |
| How painful did you find the insoles? 0 = No pain 10 = Extremely painful | **RFS** | 1.4 ± 0.8 | 2.4 ± 1.8 | 2.1 ± 1.4 | 2.1 ± 1.3 |
| | **FFS** | 1.1 ± 1.1 | 1.7 ± 1.6 | 2.0 ± 1.3 | 2.6 ± 2.3 |
| | **average** | 1.3 ± 0.9 | 2.1 ± 1.7 | 2.1 ± 1.3 | 2.4 ± 1.8 |
| Borg's Rating of Perceived Exertion | **RFS** | 6.9 ± 0.8 | 9.8 ± 2.0 | 9.9 ± 0.8 | 11.3 ± 1.4 |
| | **FFS** | 6.8 ± 1.2 | 10.6 ± 1.3 | 10.6 ± 1.3 | 12.0 ± 1.2 |
| | **average** | 6.8 ± 1.0 | 10.1 ± 1.7 | 10.2 ± 1.4 | 11.6 ± 1.3 |
| Did the insoles cause you to alter your technique? 0 = No, 1 = Yes | **RFS** | 0.1 ± 0.4 | 0.0 ± 0.0 | 0.1 ± 0.3 | 0.0 ± 0.0 |
| | **FFS** | 0.0 ± 0.0 | 0.3 ± 0.5 | 0.3 ± 0.5 | 0.3 ± 0.5 |
| | **average** | 0.1 ± 0.3 | 0.2 ± 0.4 | 0.2 ± 0.4 | 0.1 ± 0.3 |

No statistically significant results were identified. Rearfoot strike (RFS), Forefoot strike (FFS)

*Study Limitations*

The authors acknowledge that the study has a number of limitations. First, we were restricted to assessing the effect of arch compression at a relatively slow running speed. It is predicted that the energetic saving resulting from arch elastic energy storage and return is larger during faster level running, and this might also be the case for incline running.

It is also important to note that the plantar fascia can undergo strain and energy storage due to motion at the MTP joint independent of arch compression. In addition, restricting arch compression may alter intrinsic foot muscle function, which Kelly et al. [6,7] have recently shown are capable of controlling arch position and are active in running.  Despite these possible factors, measurements of MTP joint motion and net moments displayed minimal difference between the minimal shoe only and FAI/HAI trials (unpublished data [8]), suggesting they may have had a small effect.  Moreover, intrinsic foot muscles likely contribute relatively little to the overall changes in energy cost given their small size (~ 45g [9]).

A number of limitations also existed in estimating arch compression and elastic energy. A skin mounted marker placed on the navicular tuberosity was used to determine arch compression. While

this is one of the most commonly used methods for assessing arch compression (e.g. [6,10-12]), it is not without errors and may have under-estimated bony midfoot motion [13].

Our model of arch elastic energy storage/release depended on an estimate of arch compressive load from inverse dynamic calculations of ankle joint moments and Achilles tendon force that are both subject to error. The Achilles tendon moment arm was measured in a static standing position as the perpendicular distance from the mid-point of the lateral malleoli to the Achilles tendon [14]. While this measurement was performed by the same assessor it is understood that this method may differ from imaging-based measurements and does not represent the active moment arm of the Achilles during locomotion [15]. Furthermore, we assumed the hysteresis of the arch spring to be 22% based on the experimental data of Ker et al. [3], although the actual hysteresis may differ between participants. Nevertheless, differences in the average hysteresis would only change the magnitude of the predicted elastic energy return (and the subsequent predicted energy cost of locomotion), but the relative differences between conditions would remain similar. Thus, although it serves as a simple estimation of energy storage in the arch, the modelled load-displacement curve may lead to errors in estimating arch elastic energy storage with a very high degree of accuracy.

Our modelled prediction of the metabolic effect of restricting arch compression and spring function depended on assumptions regarding the efficiency of performing positive locomotor mechanical work. We used an efficiency of 25%, which represents a value close to the theoretical maximal efficiency of muscle performing positive work [16]. These predictions therefore assume that the mechanical work performed to replace the lost elastic arch work is done by positive muscle fiber work, and that this additional muscle fiber work dictates the changes observed in metabolic cost. Although these assumptions are reasonable, it is important to note that the lost arch elastic work may have been substituted, to an extent, with an increase in elastic work performed at other joints. This would necessitate an increase in force in the muscle-tendon-units generating the elastic work,

which would exact a metabolic cost. In this case the increase in metabolic cost may not be dictated solely by increases in muscle fibers producing mechanical work at a constant efficiency. Therefore, as a secondary approach to predicting the increase in metabolic cost of locomotion arising from restricting arch compression we used a computed locomotor mechanical efficiency of performing the positive mechanical work in the minimal shoe-only conditions (Equations S4 and S5). This prediction does not necessitate that the additional metabolic energy expenditure is due strictly to additional muscle fiber work alone, but rather results from a proportional increase in the combined mechanical costs that dictate the locomotor efficiency in the level running, incline running or walking conditions (e.g. energy expended in isometric contractions, muscle fiber work, etc.). Modelled metabolic cost predictions from the locomotor mechanical efficiency showed the same overall findings as those using a constant efficiency of 25% (See Table S2). Finally, it is also possible that muscle fiber efficiency was different from the 25% used in this study. If average muscle fiber efficiency was different this would alter the magnitude of the predicted increases in metabolic energy cost, although the relative differences between conditions would be expected to remain similar.

We acknowledge the possibility that our observations reflect a more general effect of altering gait mechanics with an arch-restricting insole. However, notwithstanding the aforementioned limitations, there are several factors that lead the authors to dispute this interpretation. First, while the difference in arch compression was nearly two-fold between the FAI and HAI, both insoles resulted in a similar loss of arch elastic energy storage and exhibited a non-significant difference in metabolic cost (Figures 1 & 2 in the main article). These data suggest that the increase in metabolic cost of running while wearing the insoles is not simply a systematic effect of altering arch kinematics *per se*, but instead an effect of the non-linear arch elastic energy storage/return. Secondly, the fact that there was no change in energy cost during both the FAI incline running and walking conditions strengthens the interpretation that the energetic changes observed in level running are primarily

attributed to alterations in the arch spring mechanics. If other general modifications such as co-contraction, instability, intrinsic foot muscle activity [7], cushioning [17] or discomfort [18] were the primary factors leading to the increase in metabolic cost after restricting arch compression, it would be expected that they would also elevate metabolic costs during incline running and possibly also during walking. Finally, peak ankle eversion during level running was unaffected by the insoles (Figure S3) and there were small differences in total limb mechanical work between the insole and minimal shoe only conditions (Table 1 main article).

**Orthotic clause**

The authors would like to note that the foot insoles used in this study do not represent conventional prescription practices by health practitioners for symptomatic individuals in a clinical setting, but rather a tool for experimentally testing our hypotheses.

**References**

1 Stearne, S. M., Alderson, J. A., Green, B., Donnelly, C. J. & Rubenson, J. Joint kinetics in rearfoot versus forefoot running: Implications of switching technique. *Med Sci Sports Exerc* **46**, 1578–1587, doi:10.1249/MSS.0000000000000254 (2014).

2 Stefanyshyn, D. & Nigg, B. M. Mechanical energy contribution of the metatarsophalangeal joint to running and sprinting. *J Biomech* **30**, 1081-1085 (1997).

3 Ker, R. F., Bennett, M. B., Bibby, S. R., Kester, R. C. & Alexander, R. M. The spring in the arch of the human foot. *Nature* **325**, 147-149 (1987).

4 Donelan, M. J., Kram, R. & Kuo, A. D. Simultaneous positive and negative external mechanical work in human walking. *J Biomech* **35**, 117-124, doi:10.1016/S0021-9290(01)00169-5 (2002).

5 Cheng, B. *et al.* A new approach for the determination of ventilatory and lactate thresholds. *Int J Sports Med* **13**, 518-522, doi:10.1055/s-2007-1021309 (1992).

6 Kelly, L. A., Cresswell, A. G., Racinais, S., Whiteley, R. & Lichtwark, G. Intrinsic foot muscles have the capacity to control deformation of the longitudinal arch. *J R Soc Inter* **11**, 20131188, doi:10.1098/rsif.2013.1188 (2014).

7 Kelly, L. A., Lichtwark, G. A. & Cresswell, A. G. Active regulation of longitudinal arch compression and recoil during walking and running. *J R Soc Interface* **12**, 20141076, doi:10.1098/rsif.2014.1076 (2015).

8 McDonald, K. A. *The role of arch compression and metatarsophalangeal joint dynamics in modulating plantar fascia strain in running* (The University of Western Australia, UnPublished, 2014).

9 Kura, H., Luo, Z. P., Kitaoka, H. B. & An, K. N. Quantitative analysis of the intrinsic muscles of the foot. *The Anatomical record* **249**, 143-151 (1997).

10 Ferber, R. & Benson, B. Changes in multi-segment foot biomechanics with a heat-mouldable semi-custom foot orthotic device. *J Foot Ankle Res* **4**, 1-8, doi:10.1186/1757-1146-4-18 (2011).

11 Perl, D., Daoud, A. & Lieberman, D. Effects of footwear and strike type on running economy. *Med Sci Sports Exerc* **44**, 1335–1343, doi:10.1249/MSS.0b013e318247989e (2012).

12 Boyer, E. R., Ward, E. D. & Derrick, T. R. Medial longitudinal arch mechanics before and after a 45-minute run. *J Am Podiatr Med Assoc* **104**, 349-356, doi:10.7547/0003-0538-104.4.349 (2014).

13 Nester, C. *et al.* Foot kinematics during walking measured using bone and surface mounted markers. *J Biomech* **40**, 3412-3423, doi:org/10.1016/j.jbiomech.2007.05.019 (2007).

14 Lee, S. S. M. & Piazza, S. J. Inversion–eversion moment arms of gastrocnemius and tibialis anterior measured in vivo. *J Biomech* **41**, 3366-3370, doi:org/10.1016/j.jbiomech.2008.09.029 (2008).

15 Maganaris, C. N., Baltzopoulos, V. & Sargeant, A. J. Changes in achilles tendon moment arm from rest to maximum isometric plantarflexion: *In vivo* observations in man. *J Physiol* **510**, 977—985 (1998).

16 Woledge, R. C., Curtin, N. A. & Homsher, E. Energetic aspects of muscle contraction. *Monogr Physiol Soc* **41**, 1-357 (1985).

17 Franz, J. R., Wierzbinski, C. M. & Kram, R. Metabolic cost of running barefoot versus shod: Is lighter better? *Med Sci Sports Exerc* **44**, 1519-1525, doi:10.1249/MSS.0b013e3182514a88. (2012).

18 Nigg, B. M. The role of impact forces and foot pronation: A new paradigm. *Clin J Sport Med* **11**, 2-9 (2001).
